# COL1A1 novel splice variant in osteogenesis imperfecta and splicing variants review: A case report

**DOI:** 10.3389/fsurg.2022.986372

**Published:** 2022-09-15

**Authors:** Michella Dirani, Victor D. Cuenca, Vanessa I. Romero

**Affiliations:** School of Medicine, Universidad San Francisco de Quito, Quito, Ecuador

**Keywords:** osteogenesis imperfecta, splicing, Ecuador, COL1A1, collagen

## Abstract

**Background:**

Osteogenesis imperfecta (OI) is a rare heterogeneous genetic disorder commonly autosomal dominant with variants in the COL1A1 and COL1A2 genes. It is characterized by bone fragility and deformity, recurrent fractures, blue sclera, dentinogenesis imperfecta, short stature, and progressive deafness.

**Case presentation:**

We present a novel splicing mutation in the COL1A1 gene (c.2398-1G > C) in a 6-year-old Ecuadorian girl with fractures after light pressure and blue sclera. We identified the pathogenic variant, performed a literature review of splice variants, and recognized their location in the COL1A1 functional domains.

**Conclusion:**

We describe the first clinical description of a patient with OI type 1 caused by a splice variant in intron 34 of COL1A1 gene and identify that most of them are localized in the triple-helical region domain. We suggest that the splice variant in signal peptide, von Willebrand factor type C, and nonhelical regions maintain their functionality or that individuals affected with severe cases die early in development and are not reported.

## Introduction

Osteogenesis imperfecta (OI) known as brittle bone disease is a rare heterogeneous genetic disorder of connective tissue with an incidence of 1 in 15,000–20,000 births (1). Clinically it is characterized by bone fragility, deformity, recurrent fractures, blue sclera, dentinogenesis imperfecta, short stature, and progressive deafness (1). Under clinical and radiological parameters, the disease is classified according to the Sillence classification in four types: I (mild) and IV (moderate deforming), frequent but less severe; II (lethal) and III (severe deforming), less frequent but more lethal (2).

OI is mostly autosomal dominant and 95% of cases result from mutations in COL1A1 and COL1A2 that code for type I collagen alpha chains, α1 and α2, respectively (1). Autosomal recessive cases result from mutations in the CRTAP and LEPRE1 genes (3). COL1A1variants include missense, nonsense, frameshift, and splice site (4). RNA splicing is indispensable for protein synthesis. The RNA transcript (pre-mRNA) requires introns being spliced and the exons bound to form mRNA (1). The splicing process is performed by the spliceosome, which consists of five uridine-rich ribonucleoproteins and more than 200 associated proteins (4). Inappropriate splicing fails to remove introns or removes necessary exons resulting in an abnormal type I collagen chain (5).

Here, we report a patient with fractures after light pressure and blue sclera, identify the pathogenic variant, performed a literature review of splice variants, and recognized their location in the COL1A1 functional domains. Most of articles focus on nonsense and missense variants and splice variant information are not available.

## Case report

### Patient information and therapeutic interventions

**Figure 1:**
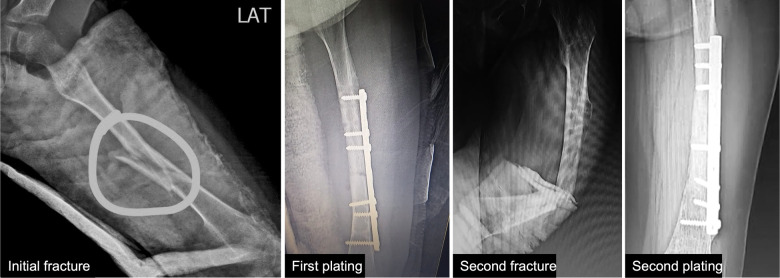
Patient’s x-rays. First x-ray shows the initial left femoral shaft fracture. Second x-ray shows the first plating fixation. Third x-ray shows the fracture after a slight twist of the leg. Fourth x-ray shows the second plating fixation.

We report a 6-year-old female who came to the genetic outpatient clinic for multiple consecutive fractures. She was born by vaginal delivery from nonconsanguineous parents with no complications and achieved all developmental milestones without delay. These include fine motor, gross motor social-emotional, and behavioral, language, and cognitive milestones (6). At 6 months, she presented hip dysplasia and bilateral valgus cavus that was treated with an orthopedic diaper. In June 2021, she fell from her height and suffered a left femoral shaft close fracture that required surgery and plating nailing placement (Figure 1 and Supplementary Figure S1). Eight months later, the surgeon removed the plating. Fifteen days later, the girl slightly twisted her left leg and fractured her left femur again. The pediatrician ordered additional imaging studies and ruled out malignancy. Her physical examination revealed blue sclera without coloboma and hypermobility in her hands and feet. Her family history information is relevant for blue sclera but no fractures in her mother.

### Molecular analysis

We ordered the Osteogenesis Imperfecta and Bone Fragility Panel that analyzes 67 genes and identified a heterozygous G > C c.2398-1 (splice acceptor) pathogenic variant in intron 34 of COL1A1. The variant alters splicing with a frequency of 0 in The Genome Aggregation Database (gnomAD) and in The Exome Aggregation Consortium (ExAC) databases. The splice variant was confirmed by Sanger sequencing.

### Review of splicing variants

**Figure 2:**
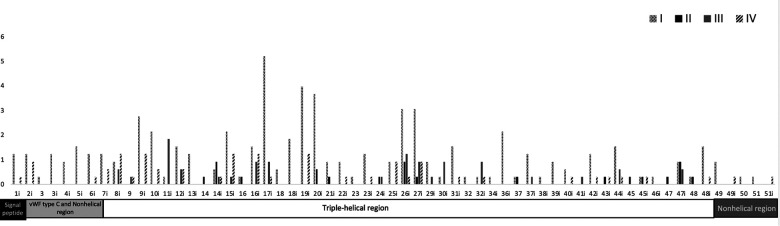
Frequency of the location of OI splice variants and COL1A1 domain distribution. Upper region: location of the type of OI splice variants along the COL1A1 introns and exons. OI is classified according to the Sillence classification in four types: I (mild) and IV (moderate deforming), frequent but less severe; II (lethal) and III (severe deforming), less frequent but more lethal. Most splice variants are located in intron 17. Black and white pattern = type I, black = type II, gray = type III, and oblique black = type IV. Lower region: functional domains along COL1A1 introns and exons. Most splice variants are located in the triple-helical region. Black square = signal peptide, light gray rectangle = von Willebrand factor type C and nonhelical region (N-terminal), white rectangle = triple-helical region, and dark gray rectangle = nonhelical region (C-terminal).

We reviewed all cases of splicing variants reported in PubMed until June 2022 and filtered the case reports that include the splice variant location and the OI type. We identified 336 splicing variants, most of them were OI type I and located in intron 17. Most of the splice variants were located in introns 28, 33, 35, and 50 and in exon 49 (Figure 2, Supplementary Tables S1, S2). OI type I was located mostly in intron 17 and 19; type 2 in intron 14, 26, and 47; type 3 in intron 11 and 6; and type 4 in intron 8, 9, 15, 16, and 19. The most intron splice variants were located in introns near exons that become the triple-helical region domain (Supplementary Table S2). Splice variant mostly caused OI type 1. Intron 17 includes the most single nucleotide variants (mostly G > C/A) and reported 17 type 1, 3 type 3, and 1 type 4 case. Most of single nucleotide variants in OI type 2 are A > C and type 3 G > C (Supplementary Table S1).

## Discussion

In this case report, we describe the first clinical description of a patient with a splice variant in intron 34 in the COL1A1 gene with a systemic literature review of splice variants reported in PubMed and used the variant database by Dalgeish et al. We identified that most of the variants localize in the triple-helical region domain.

Our patient presented with two fractures after weak pressure and blue sclera, and neoplasia was excluded. The sequencing panel reported a splice variant in intron 34. A splice variant in exon 34 was reported by Lindahl et al.. and a study in Sweden mentioned that one case had a OI type IA and did not include the clinical description (7). Our case is the first report of a c.2398-1G > C variant, is a OI type I, and the mother of the patient has blue sclera and lacks medical history of fractures.

The differential diagnoses of OI include diseases related to bone fragility (8). Rickets was excluded due to a normal alkaline phosphatase level, normal sclera and audition involvement, or deafness. Osteomalacia is characterized by bone pain and fractures (8). Other disorders to consider include Bruck's syndrome (congenital contractures involving knee or ankle joints), panostotic fibrous dysplasia (cystic lesions in all bones), juvenile Paget’s disease (elevated alkaline phosphatase levels), or juvenile idiopathic osteoporosis (9).

The COL1A1 gene is located in chromosome 17, includes 17,554 bp, and produces pro-alpha 1 collagen chains. Type 1 collagen is the most common type of collagen in the body and is part of bones, skin, tendons, and sclera (10). Type 1 collagen contains two pro-alpha 1 chains and one pro-alpha 2 chain. The functional domains include signal peptide (exon 1), von Willebrand factor type C and nonhelical region (exon 2–6), triple-helical region (exon 7–48), and nonhelical region (exon 49–52). The triple helix repeat works as an extracellular structural protein involved in the formation of connective tissue structure. Mutations in COL1A1 alter the production of type 1 collagen resulting in bone fragility, and most published articles focused on missense and nonsense variants. A splice variant is a modification in the DNA sequence in the splice site (the limit between exons and introns), which can cause the inclusion of introns, loss of exons, and alteration of the protein coding sequence, disrupting RNA splicing (11). We found that most of the splice variants are located in the triple-helical region and are mostly OI type I (Figure 2, Supplementary Tables S1, S2). The triple-helical region includes a series of repeated regions and additional introns, or lesser exons allow for the diverse clinical types. The signal peptide, von Willebrand factor type C and nonhelical region, and nonhelical regions have OI type I (mild) and IV (moderate). We suggest that the splice variant in these regions maintain the functionality or that individuals affected with severe cases die early in development and are not reported.

### Examples of different phenotypes by different splice variants

Splice variants in the same gene can cause different phenotypes. Errors during splicing leads to alterations in the reading frame by including introns or excluding exons. For example, the variant c.3718-2477C > T in CFTR that is reported in patients with cystic fibrosis produces a stop codon, creates a nonfunctional protein, and present a mild phenotype (12). Likewise, most hemophilia B patients with splicing mutations correspond have from to severe moderate to moderate severe phenotypes, consequence of the aberrant splicing that conditions the expression of the functional protein. The c.520 + 13A > G mutation in intron 5 shows moderate mild to mild moderate phenotypes (13). In addition, same substitution at the same location of the DMD gene can cause different clinical manifestations on the patient. Mutation c.3277 + 1G > A occurring in intron 25 completely removes exon 25 resulting in a mild Becker muscular dystrophy, while mutation c.6439 + 1G > A in intron 45 results in the inclusion of a shorter exon 45 leading to a severe form of Duchenne muscular dystrophy (12).

## Conclusion

We described the first clinical description of a patient with OI type 1 caused by a splice variant in intron 34 of COL1A1 gene, performed a literature review of splice variants, and identified that most of them localize in the triple-helical region domain.

## Data Availability

The datasets presented in this article are not readily available because of ethical/privacy restrictions. Requests to access the datasets should be directed to the corresponding author.

## References

[1] Barbirato C Almeida MG Milanez M Sipolatti V Rebouças MRGO Akel AN A novel COL1A1 gene-splicing mutation (c.1875+1G > C) in a Brazilian patient with osteogenesis imperfecta. Genet Mol Res GMR. (2009) 8(1):173–8. 10.4238/vol8-1gmr563 19283684

[2] Lin Y Li X Huang X Zheng D Liu Y Lan F Hybrid minigene splicing assay verifies the pathogenicity of a novel splice site variant in the COL1A1 gene of a Chinese patient with osteogenesis imperfecta type I. Injury. (2019) 50(12):2215–9. 10.1016/j.injury.2019.10.033 31653500

[3] Ju M Bai X Zhang T Lin Y Yang L Zhou H Mutation spectrum of COL1A1/COL1A2 screening by high-resolution melting analysis of Chinese patients with osteogenesis imperfecta. J Bone Miner Metab. (2020) 38(2):188–97. 10.1007/s00774-019-01039-3 31414283

[4] Li L Cao Y Zhao F Mao B Ren X Wang Y Validation and classification of atypical splicing variants associated with osteogenesis imperfecta. Front Genet. (2019) 10:979. 10.3389/fgene.2019.00979. 31737030 PMC6832110

[5] Marini JC Forlino A Cabral WA Barnes AM San Antonio JD Milgrom S Consortium for osteogenesis imperfecta mutations in the helical domain of type I collagen: regions rich in lethal mutations align with collagen binding sites for integrins and proteoglycans. Hum Mutat. (2007) 28(3):209–21. 10.1002/humu.20429 17078022 PMC4144349

[6] Scharf RJ Scharf GJ Stroustrup A. Developmental milestones. Pediatr Rev. (2016) 37(1):25–37, quiz 38–47. 10.1542/pir.2014-0103 26729779

[7] Lindahl K Åström E Rubin CJ Grigelioniene G Malmgren B Ljunggren Ö Genetic epidemiology, prevalence, and genotype-phenotype correlations in the Swedish population with osteogenesis imperfecta. Eur J Hum Genet. (2015) 23(8):1042–50. 10.1038/ejhg.2015.81 25944380 PMC4795106

[8] Sam JE Dharmalingam M. Osteogenesis imperfecta. Indian J Endocrinol Metab. (2017) 21(6):903–8. 10.4103/ijem.IJEM_220_17 29285457 PMC5729682

[9] Rauch F Glorieux FH. Osteogenesis imperfecta. Lancet. (2004) 363(9418):1377–85. 10.1016/S0140-6736(04)16051-0 15110498

[10] PubChem. COL1A1—collagen type I alpha 1 chain (human) (2022). Available at: https://pubchem.ncbi.nlm.nih.gov/gene/COL1A1/human (Accessed January 20, 2022).

[11] Definition of splice-site variant—NCI dictionary of genetics terms—NCI (2012). Available at: https://www.cancer.gov/publications/dictionaries/genetics-dictionary/def/splice-site-variant (Accessed January 8, 2022).

[12] Anna A Monika G. Splicing mutations in human genetic disorders: examples, detection, and confirmation. J Appl Genet. (2018) 59(3):253–68. 10.1007/s13353-018-0444-7 29680930 PMC6060985

[13] Shen G Gao M Cao Q Li W. The molecular basis of FIX deficiency in hemophilia B. Int J Mol Sci. (2022) 23(5):2762. 10.3390/ijms23052762 35269902 PMC8911121

