# Supplementary figures and images for: COL1A1 novel splice variant in osteogenesis imperfecta and splicing variants review: A case report
Source: Front Surg. 2022 Sep 15;9:986372. doi: 10.3389/fsurg.2022.986372 (PMC9632975; doi:10.3389/fsurg.2022.986372)

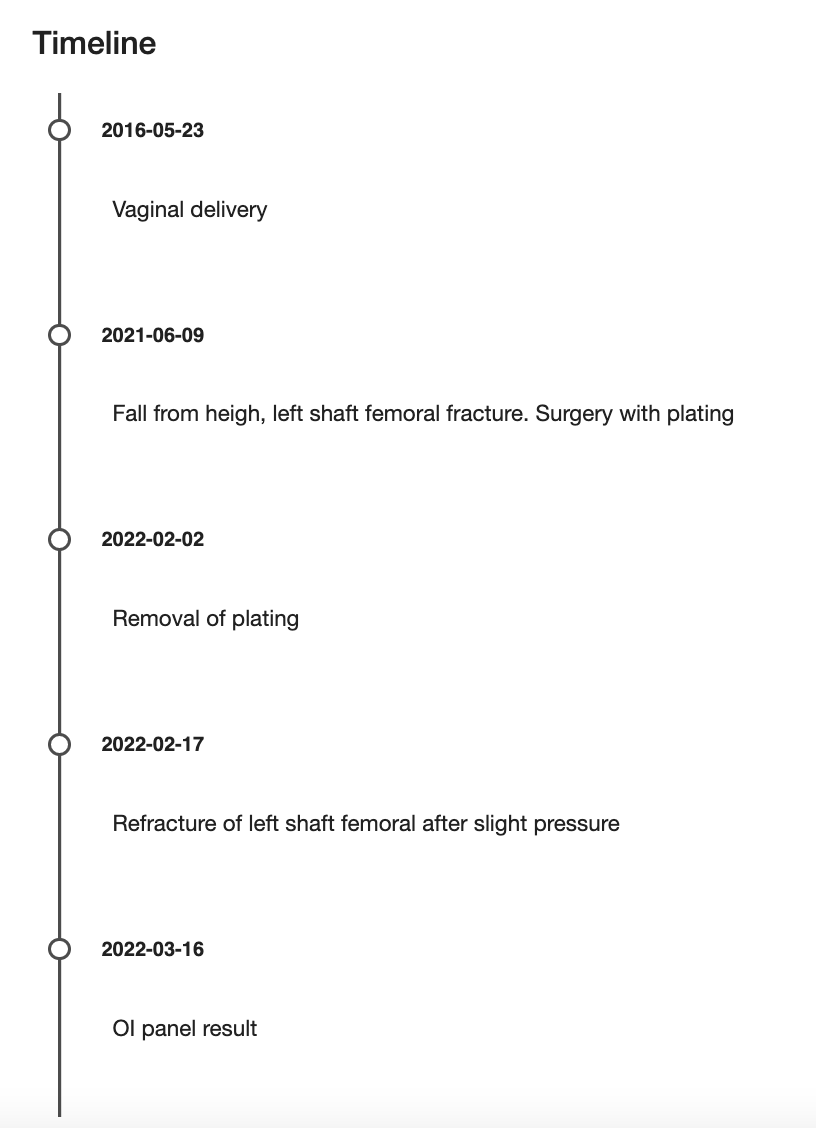

Supplement: Supplementary file 2 [file Image1.png]
